# The suppressive role of calcium sensing receptor in endometrial cancer

**DOI:** 10.1038/s41598-018-19286-1

**Published:** 2018-01-18

**Authors:** Xiaoyan Xin, Xianqin Zeng, Dilu Feng, Teng Hua, Shuangge Liu, Shuqi Chi, Qinghua Hu, Hongbo Wang

**Affiliations:** 1 Department of Obstetrics and Gynecology, Union Hospital, Tongji Medical College, Huazhong University of Science and Technology, No.1277, Jiefang Avenue, Jiangan District, Wuhan, 430022 PR China; 2 Department of Pathophysiology, School of Basic Medicine, Tongji Medical College, Huazhong University of Science and Technology, No.13, Hangkong Road, Qiaokou District, Wuhan, 430030 PR China

## Abstract

Studies have shown that calcium sensing receptor (CaSR) is involved in the progressions of several human cancers. However, the role of CaSR in endometrial cancer remains unknown. This study provides a preliminary analysis of the CaSR effect on endometrial cancer development. Ectopic CaSR expression by lentiviral transfection (CaSR-OV) in Ishikawa cells significantly increased intracellular calcium ([Ca^2+^]_i_) levels and cell apoptosis. E-cadherin and β-catenin expression and complex formation at the membrane were increased in CaSR-OV Ishikawa cells relative to control Ishikawa cells (vector). Furthermore, CaSR-OV Ishikawa cells showed a reduced invasive potential, which was attributed to E-cadherin/β-catenin complex formation. Moreover, a reduction in CaSR expression in endometrial cancer relative to normal specimens was evident by immunohistochemistry and was positively associated with E-cadherin, but not β-catenin, expression. Furthermore, VEGFR3 was significantly down-regulated in CaSR-OV Ishikawa cells. Additionally, an immunohistochemical analysis showed that VEGFR3 was significantly increased in endometrial cancer compared with the normal endometrium and was inversely correlated with CaSR expression. However, the CaSR knockdown produced the opposite effects. These findings suggest an inhibitory role for CaSR in endometrial cancer. Therefore, reduced CaSR expression may be a suitable explanation and valuable predictor for endometrial cancer progression.

## Introduction

Endometrial cancer is predominant in developed countries and is among the top three most common reproductive tract malignancies worldwide. An estimated 54,870 new cases of endometrial cancer with 10,170 deaths from this disease were predicted in the United States in 2015^1^. The five-year relative survival rates for localized-, regional- and distal-stage endometrial cancers are 95%, 68% and 18%, respectively^1^. Endometrial tumour metastasis is an important risk factor for a poor prognosis, and further studies in this field are needed to elucidate the disease mechanism.

The primary cause of death by a malignant tumour is its inherent metastatic property. Tumour metastasis is a complex, multi-step pathological process that involves the potential loss of the molecules ensuring cell-cell adhesion in a metastatic tumour, the escape of the tumour cells into lymphatic and blood vessels, their subsequent travel to distal organs, and independent growth into new tumours. Although there are numerous attempts to explain the metastatic process, including the cancer stem cell theory, the epithelial-mesenchymal transition (EMT), the homing process, and changes in tumour microenvironment, the precise underlying mechanisms are not fully understood; this has presented a challenge for developing anti-metastatic therapeutic modalities^2–4^.

Numerous studies have indicated that extracellular CaSR plays a critical role in tumour development. CaSR is a Class C G-protein-coupled receptor that senses and responds to fluctuations in circulating Ca^2+^ primarily by regulating parathyroid hormone (PTH) secretion^5^. CaSR is physiologically activated by polyamines, polyvalent cations, amino acids and pH changes prior to its involvement in regulating physiological and pathological processes that include ion channel activity, ionic homeostasis, hormone secretion, inflammation, proliferation, apoptosis, differentiation, cell adhesion, chemosensitivity, and others^6–8^. However, there are no published studies on the role of CaSR in endometrial cancer.

The purpose of this study was to characterize the role of CaSR in endometrial cancer and to understand the underlying mechanisms behind CaSR-mediated regulation of apoptosis, invasion and metastasis with the goal of uncovering a new anticancer target for endometrial cancer. Our results showed clear CaSR expression in an endometrial cancer cell line and decreased expression in endometrial cancer specimens. Moreover, excessive CaSR expression contributed towards attenuating cell proliferation, invasion, cell adhesions and VEGFR3 in endometrial cancer.

## Results

### CaSR expression induces a rise in [Ca2+] in response to elevated [Ca2+]o

**Figure 1:**
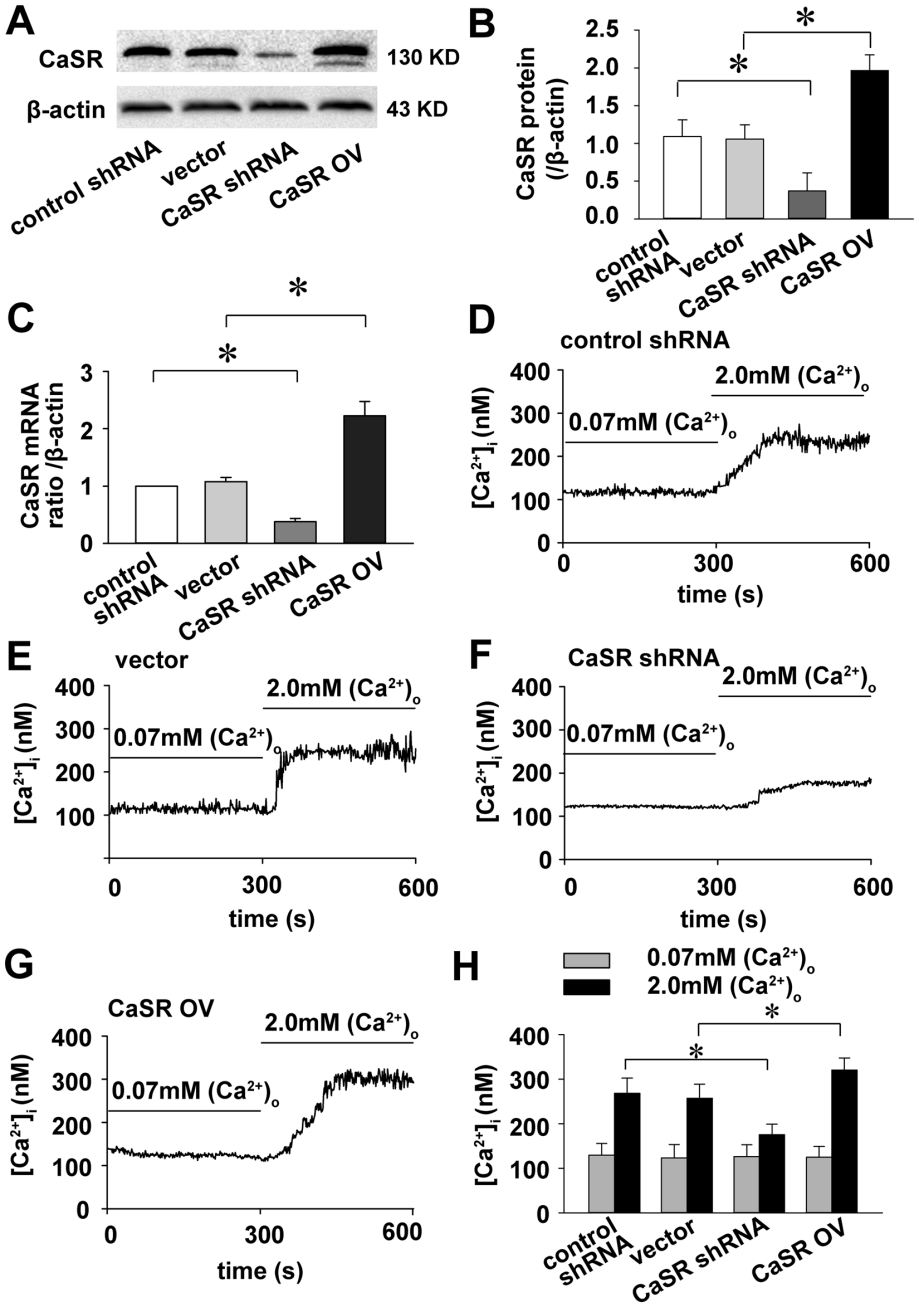
Differential CaSR expression by transfection affects the acute [Ca2+]i response to elevated [Ca2+]o. CaSR was expressed in Ishikawa cells and altered by transfection with a shRNA-CaSR or cDNA-CaSR lentivirus as described. The Western blot (n = 4) and real-time PCR (n = 3) results show that the short hairpin RNA- and cDNA-mediated alterations succeeded in knocking down and overexpressing CaSR in Ishikawa cells, respectively (**A–C**; *p < 0.05). Cells were exposed to medium containing 0.07 mM [Ca2+]o and switched to 2.0 mM [Ca2+]o to verify CaSR function. A representative curve of the [Ca2+]i response to the converted concentration [Ca2+]o is shown for control shRNA-transfected cells, empty vector-transfected cells (without cDNA), CaSR shRNA-transfected cells and CaSR-OV-transfected cells, respectively (**D**–**G**). The significantly decreased and increased [Ca2+]i levels were consistent with the CaSR knockdown and overexpression, respectively (**H**; cells number = 11 *p < 0.05).

To explore whether CaSR was expressed in endometrial cancer, a Western blot analysis was performed; it showed that CaSR was expressed in Ishikawa cells. CaSR expression was successfully knocked down or enhanced when Ishikawa cells were transfected with a shRNA-CaSR or cDNA-CaSR lentivirus, respectively; this was validated by Western blot and real-time PCR (Fig. 1A–C).

To determine whether the CaSR protein was functional, Ishikawa cells were exposed to a rapid increase in the [Ca2+]o concentration (from 0.07 to 2.0 mM) each 5 min, and a [Ca2+]i measurement was conducted. As shown in Fig. 1D and F, the [Ca2+]o increase from 0.07 to 2 mM resulted in an increased [Ca2+]i for the control shRNA group, and a small increase was detected in the shRNA-CaSR group. The [Ca2+]i levels was depressed by 63.95% when CaSR was silenced (Fig. 1H). Conversely, the [Ca2+]o increase from 0.07 to 2 mM increased [Ca2+]i for the CaSR OV cells compared with the control vector cells (Fig. 1E,G). The [Ca2+]i levels was increased by 43.79% when CaSR was overexpressed. CaSR overexpression induces a rise in [Ca2+] in response to elevated [Ca2+]o (Fig. 1H). Thus, Ishikawa cells sense the elevated [Ca2+]o through CaSR-mediated [Ca2+]i signalling.

### CaSR activation decreases cell viability and invasion and induces apoptosis

**Figure 2:**
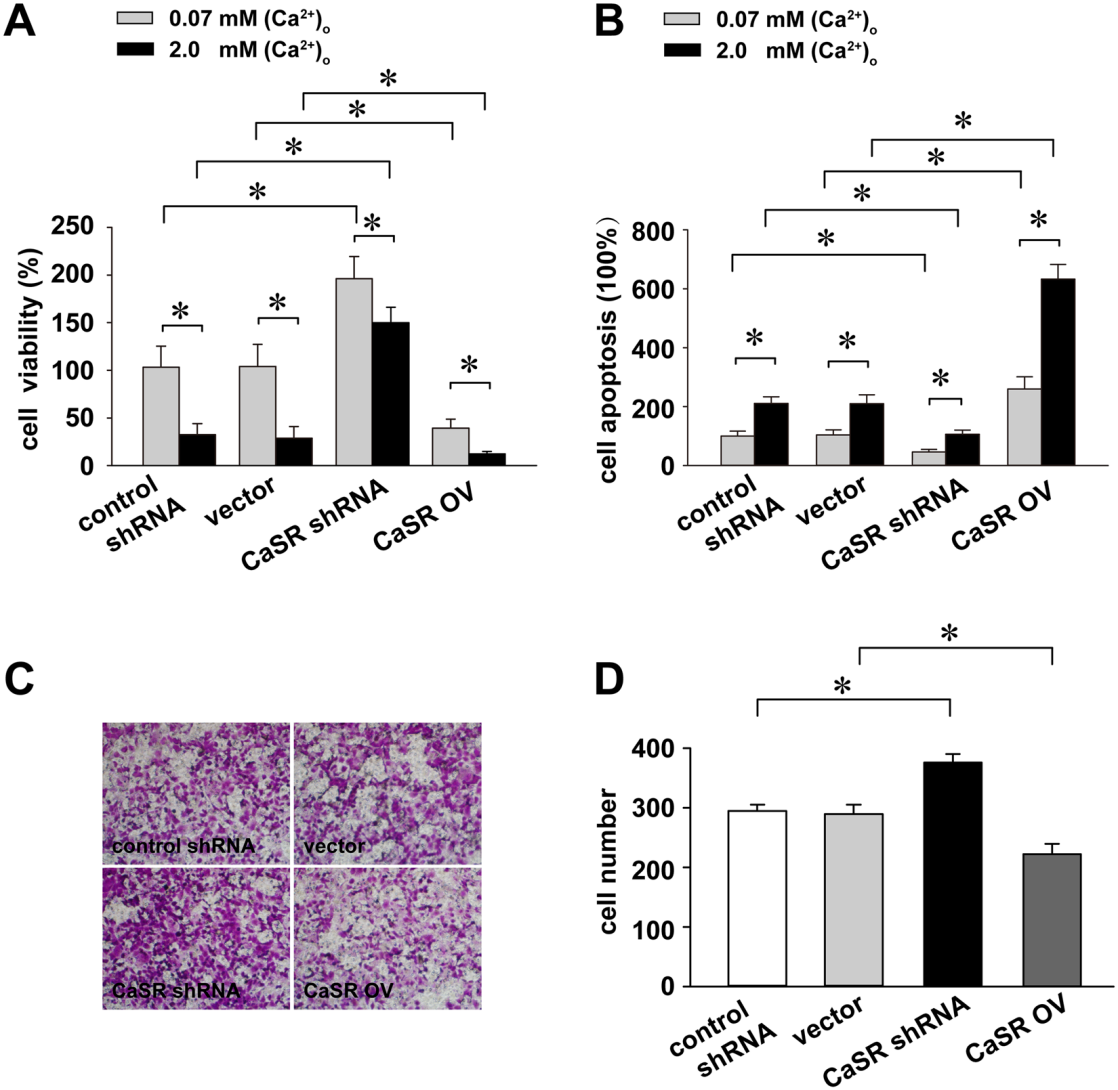
CaSR activation induces apoptosis but suppresses the invasiveness of Ishikawa cells. Ishikawa cells were transfected with a shRNA-CaSR or cDNA-CaSR lentivirus, followed by treatments with different [Ca2+]o concentrations for 48 h. After the treatments, cell viability and apoptosis were measured using MTT assays (**A**) and the ELISA cell death detection kit (**B**), respectively. Each experiment was repeated three times. The invasiveness of the CaSR-suppressed Ishikawa cells was measured using the Transwell invasion assay (**C**–**D**). *p < 0.05.

Cell counting kit-8 assays were used to determine the effect of CaSR on Ishikawa cell viability. As shown in Fig. 2A, the CaSR knockdown significantly increased cell survival (1.77-fold), whereas cell survival decreased following CaSR overexpression (3.77-fold) relative to the corresponding control group. Furthermore, high [Ca2+]o (2 mM) reduced cell viability, which was independent of the CaSR expression status. Moreover, CaSR silencing significantly decreased apoptosis, but apoptosis was increased relative to the corresponding control group when CaSR was overexpressed (Fig. 2B). Consistently, high [Ca2+]o (2 mM) induced apoptosis, which was independent of the CaSR expression status. The data indicate that CaSR and Ca2+promote Ishikawa cell apoptosis; the function of Ca2+may be only partially dependent on CaSR expression.

We performed Transwell cell invasion assays to examine whether CaSR affected the invasiveness of the Ishikawa cell line. The results revealed that CaSR silencing significantly increased invasion by Ishikawa cells, while CaSR overexpression suppressed it (Fig. 2C,D). Thus, CaSR suppressed the invasiveness of Ishikawa cells.

### CaSR activation induces E-cadherin/β-catenin complex formation

**Figure 3:**
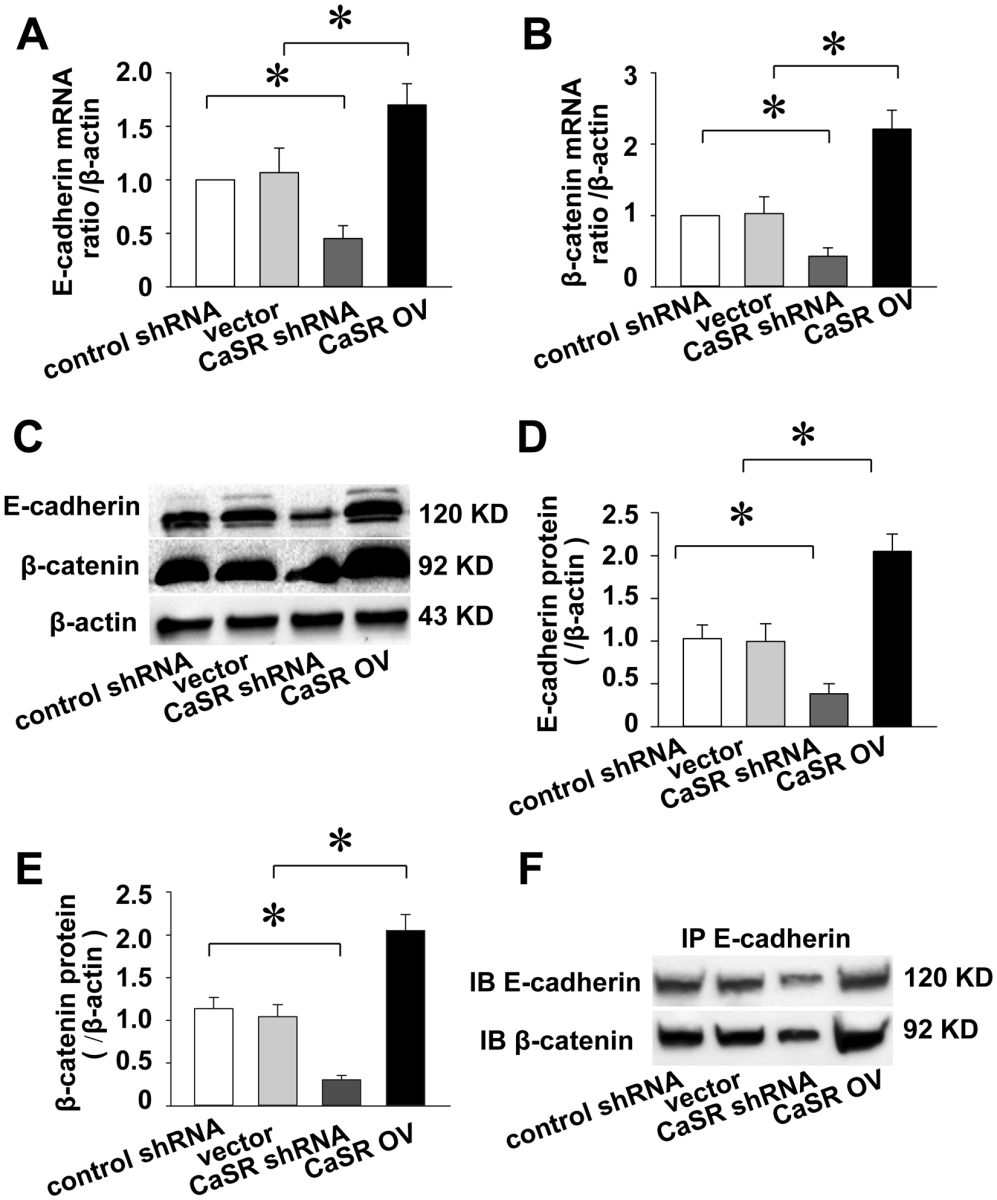
CaSR expression induces E-cadherin/β-catenin expression and complex formation the adherens junction of Ishikawa cells. The real-time PCR (n = 3) and Western blot (n = 4) results show the effects of CaSR on the expression and amplification of E-cadherin (**A**,**C**,**D**; *p < 0.05) and β-catenin (**B**,**C**,**E**; *p < 0.05). Co-immunoprecipitation of E-cadherin and β-catenin show that in cells overexpressing the CaSR there is more E-cadherin/β-catenin complex (n = 3) (**F**; *p < 0.05).

A previous study revealed an important association between CaSR and E-cadherin-mediated cell-cell adhesions in keratinocytes^9^. The role of CaSR in the intercellular adherens junctions of endometrial tumours remains unclear. Real-time PCR was performed to assess whether abundant CaSR levels altered the transcript levels of E-cadherin and β-catenin; it showed that the E-cadherin and β-catenin transcript level changes were consistent with those of the CaSR transcript in Ishikawa cells (Fig. 3A,B). The Western blotting results further proved the above phenomenon (Fig. 3C–E). This suggested that CaSR may be involved in regulating intercellular adhesion.

To validate this hypothesis, we performed co-immunoprecipitations to verify whether CaSR affected the abundance of E-cadherin/β-catenin complex. The co-immunoprecipitation assays demonstrated that CaSR inhibition decreased E-cadherin/β-catenin complexes, whereas CaSR overexpression had the opposite effect (Fig. 3F). Consequently, CaSR overexpression increased the E-cadherin/β-catenin complexes in the Ishikawa cell adherens junctions.

**Figure 4:**
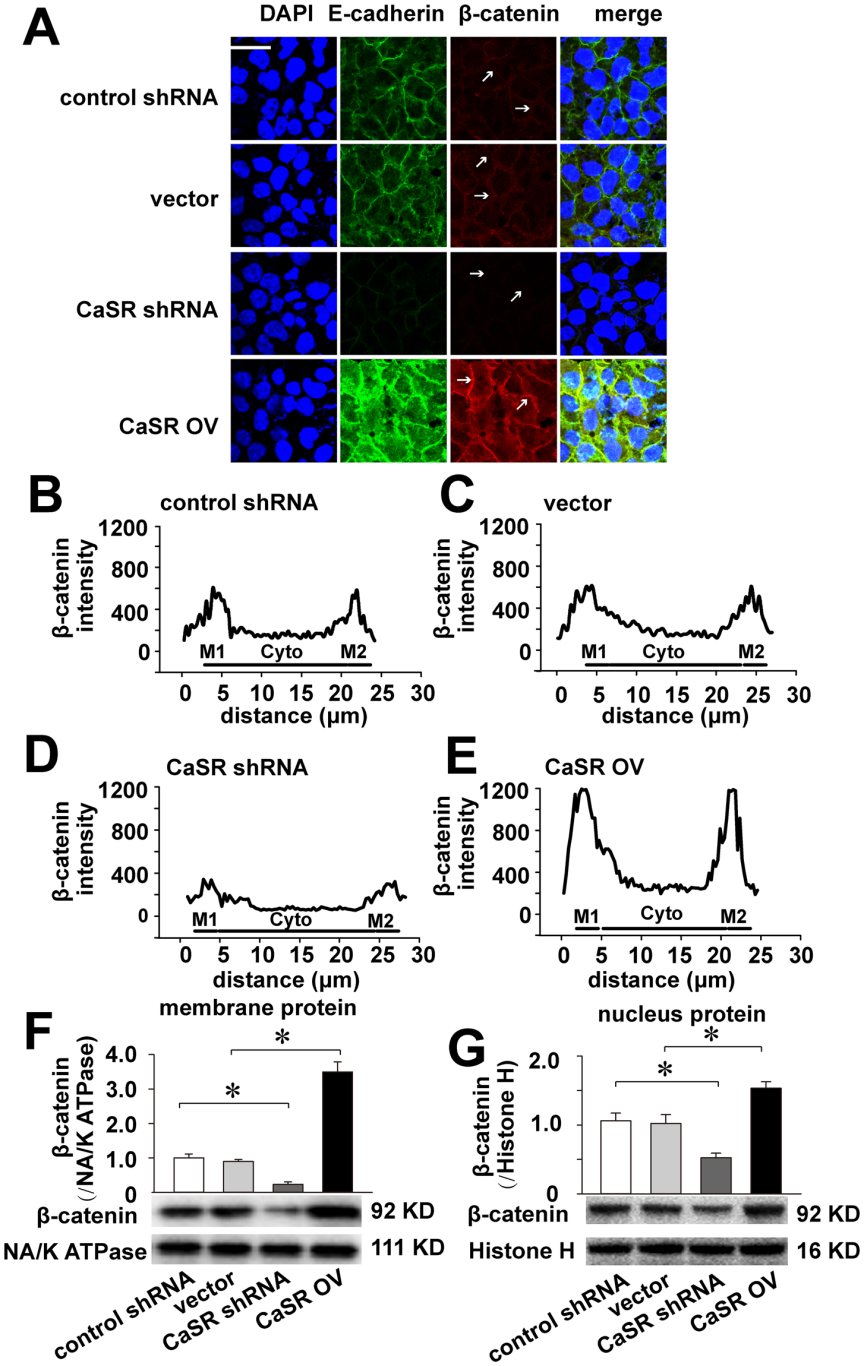
CaSR increases E-cadherin and β-catenin at the plasma membrane. Immunostaining was performed for E-cadherin and β-catenin in the control shRNA, vector, shRNA-CaSR and over-expression CaSR Ishikawa cell groups. Ishikawa cells were stained with a primary antibody against E-cadherin and a monoclonal antibody against β-catenin, followed by the appropriate FITC (Green)- or CY3 (Red)-conjugated secondary antibody. Fluorescent signals were observed by confocal microscopy. (**A**, n = 5; Bars: 20 μm). The fluorescence intensities of β-catenin were quantified along the white arrows and are presented as a percentage of the intensity of the extracted fraction versus the total intensity for the control shRNA (**B**), vector (**C**), shRNA-CaSR (**D**) and CaSR overexpression groups (**E**) (n > 80 cells/group; M1 and M2, plasma membrane; Cyto, cytoplasm). The Western blot (n = 3) results show the effects of CaSR on β-catenin expression at the membrane and nucleus in Ishikawa cells (**F**,**G**). *p < 0.05.

Immunostaining was performed to locate and quantify E-cadherin and β-catenin in each treatment group. The alterations in E-cadherin and β-catenin expression were consistent with the qRT-PCR and Western blotting results. Additionally, the merged image showed the co-localization of the two proteins to the Ishikawa cell membrane (Fig. 4A). The localization of β-catenin to the cell membrane or nucleus of a cancer cell typically shows strong, visible variations. The fluorescence intensity analysis showed that changes to the β-catenin distributions at the cell membrane were synchronized with those of CaSR (Fig. 4B–E). Western blotting was performed to determine the differential expression of β-catenin in the cell membrane and nucleus. The results showed that CaSR inhibition decreased both membrane and nuclear β-catenin expression, whereas CaSR over-expression increased both membrane and nuclear β-catenin expression (Fig. 4F,G). As the two graphs show, CaSR expression appears to effect expression of β-catenin in the cell membrane stronger than in the nucleus.

### Reduced expression of CaSR is associated with E-cadherin in endometrial cancer

**Figure 5:**
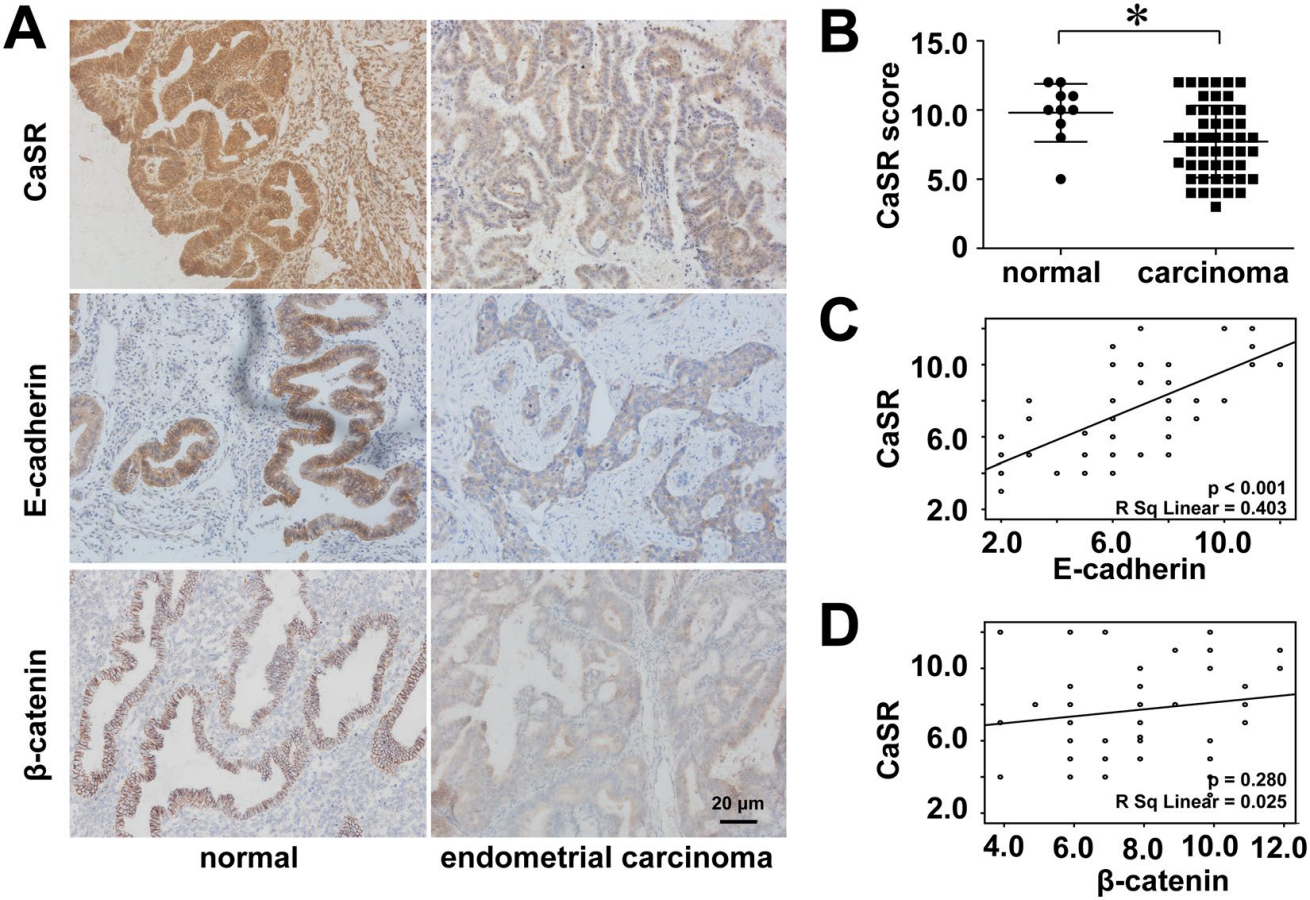
Reduced expression of CaSR and E-cadherin in endometrial cancer. Immunocytochemistry for CaSR, E-cadherin and β-catenin was performed as described; strong and weak CaSR staining are evident in the normal endometrium (n = 10) and in endometrial carcinoma (n = 50), respectively (**A**,**B**) and are in accordance with E-cadherin expression (**A**). The correlation analysis demonstrates the relationship between CaSR and E-cadherin (**C**), but not β-catenin (**D**) expression.*p < 0.05.

Immunohistochemistry was performed to determine the quantities and localizations of CaSR, E-cadherin and β-catenin in normal endometrial and tumour specimens. CaSR showed strong staining intensities in all normal endometrial tissues with no differences between the endometrial glands and stromal cells. CaSR protein expression in the endometrial epithelial cancer cells exhibited a statistically significant decrease compared to the normal group (p = 0.022) (Fig. 5A,B). A strong staining intensity was apparent for the E-cadherin protein in the normal endometrial tissues and differentiated carcinomas but not in the undifferentiated carcinomas, which showed weaker staining. Significant differences between the endometrial cancer and normal endometrial specimens were detected (p = 0.042). There was no significant difference in the staining intensity of the β-catenin protein between the endometrial cancer and normal endometrial specimens (p = 0.66) (Fig. 5A). The correlation analysis demonstrated a positive relationship between CaSR and E-cadherin expression (R^2^ = 0.403, P < 0.001), whereas there was no relationship between CaSR and β-catenin expression (R^2^ = 0.025, p = 0.280) (Fig. 5C,D). Moreover, the decreased CaSR expression correlated with several clinicopathological characteristics, including histologic grade, myometrial invasion and lymph node metastasis but not age and stage (Supplementary Table).

### CaSR activation suppresses VEGFR3 expression

**Figure 6:**
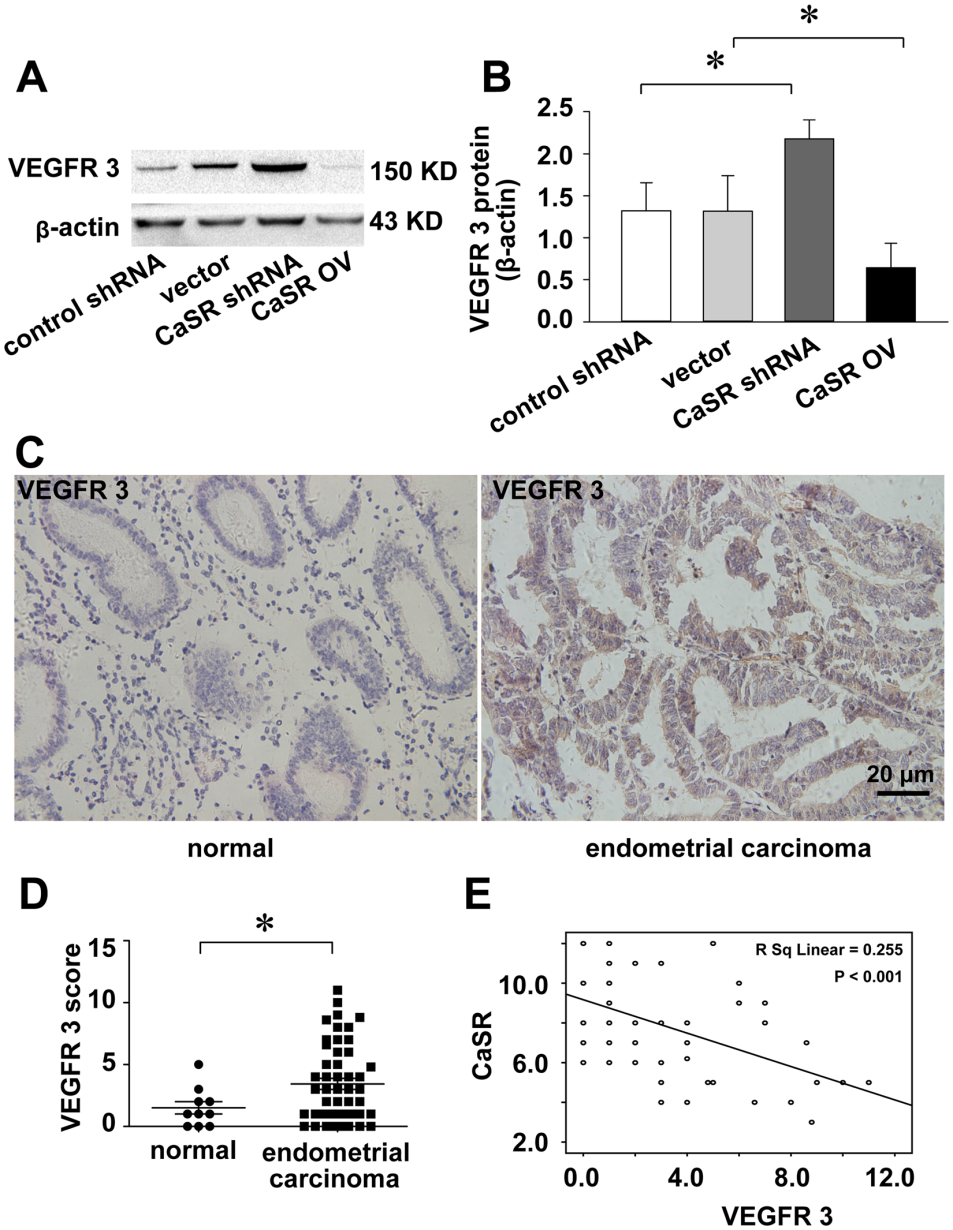
Reduced CaSR expression promotes VEGFR3 expression in endometrial cancer. The Western blot shows that CaSR expression negatively regulates VEGFR3 expression (**A**,**B**). The immunocytochemistry shows elevated VEGFR3 staining in endometrial carcinoma (n = 50) compared with the normal endometrium (n = 10) (**C**,**D**) The correlation analysis demonstrates the relationship between CaSR and VEGFR3 expression (**E**). *p < 0.05.

To our knowledge, lymphatic dissemination is a major precursor of metastasis and is a key prognostic factor for endometrial carcinoma. Surprisingly, VEGFR3, a specific lymphangiogenic factor, may be negatively regulated by CaSR, as detected by our Western blot analysis (Fig. 6A,B). In tissue specimens, VEGFR3 was significantly overexpressed in endometrial cancer relative to normal tissues and was immunolocalized to the glandular cells (P < 0.05) (Fig. 6C,D). Furthermore, CaSR expression in endometrial cancer specimens was negatively correlated with VEGFR3 (R^2^ = 0.255, P < 0.001) (Fig. 6E). Thus, CaSR may inhibit VEGFR3 expression in endometrial cancer.

## Discussion

Although the effects of Calcium, vitamin D and 1, 25(OH) 2D3 on malignancies in women remain controversial, limited (but inconclusive) evidence suggests that they may be beneficial. In this study, we highlighted calcium as a promising factor in the effort to induce apoptosis that was dependent (at least in part) on CaSR expression in endometrial cancer cells. Ectopic CaSR expression inhibited invasion, stabilized adhesion, and led to more E-cadherin/β-catenin complexes; it also decreased VEGFR3 expression in an endometrial cancer cell line. Reduced CaSR was evident in endometrial cancer relative to the normal specimen and was associated with multiple clinicopathological characteristics, including histologic grade, myometrial invasion, lymph node metastasis and E-cadherin and VEGFR3 expression. These data suggest a hypothesis that the loss of CaSR may promote endometrial cancer progression.

Several studies have demonstrated that CaSR functions as a positive regulator of tumour cell proliferation and migration. In breast cancer, CaSR activation by high calcium induces cancer cell proliferation, potentially through the EGFR-ERK1/2 axis^10,11^. The inherent mechanism is consistent with that observed in cells such as artery smooth muscle cells^12^, the axon^13^ and porcine bone marrow mesenchymal stem cells^14^. One study indicates that highly expressed CaSR induced by calciums has a promoting effect on cell migration and proliferation via its signaling pathways, like AKT, PLCγ-1, p38α and JNK/paxillin, which makes it a promising prognostic marker for predicting bone metastasis by renal cell carcinomas^15^. CaSR also promote endothelial cell migration, angiogenesis, and the formation and progression of bone metastases by functioning as a molecular sensor and promoter that targets numerous downstream factors^6,16,17^. It follows that the vital role of CaSR in tumour progression is not restricted to its participation in tumour cell proliferation; CaSR is also involved in managing subsequent distal metastases of tumours.

Numerous studies highlight CaSR as a tumour suppressor. Ectopic CaSR expression (or acute exposure to calcium) in neuroblastoma cells facilitates apoptosis, which relies on a sustained activation of ERK1/2^18^. Restoration of CaSR expression reverses the malignant phenotype, making it a candidate target for novel therapeutic strategies in colorectal cancer^19^. These studies are consistent with our findings that suggest modulation of tumour cell apoptosis by CaSR activation.

E-cadherin is recruited to maintain cell morphology by interacting with several proteins, including β-, α-and p120-catenin^20^. β-catenin may localize the nucleus to interact with transcription factors (i.e., T cell factor); it may eventually participate in regulating downstream gene transcription that further promotes tumour progression when the classic Wnt/β-catenin pathway is activated. Surprisingly, an important property of CaSR is its ability to increase intercellular adhesion in epidermal keratinocytes^9^, colorectal cancer^7,21–24^, and pancreatic cancer^25^. In agreement with these findings, our data show that CaSR expression produces similar effects in endometrial cancer. Evidence from our study suggests that ectopic CaSR overexpression improves E-cadherin and β-catenin expression and increases E-cadherin/β-catenin complex formation, which may suppress metastasis. Furthermore, several studies^22,24–26^ have shown that down-regulation of CaSR in cancers favours cell transformation. CaSR activation markedly suppresses pancreatic tumourigenesis, possibly through sodium-calcium exchanger 1-mediated Ca2+ influx; this leads to the inhibition of Wnt/β-catenin signalling in pancreatic cancer cells^25^. Stable CaSR expression in HT29, a human colon cancer cell line, promotes an epithelial-like morphology with increased E-cadherin levels and a reduced invasive potential by blocking the Wnt/β-catenin pathway, decreasing GSK-3β and Cyclin D1, and decreasing nuclear translocation of β-catenin^22^. Thus, CaSR may modulate tumour progression by suppressing E-cadherin/β-catenin complex formation. Additionally, we show that CaSR increases both membrane and nuclear β-catenin levels, and the overall effect need further analysis.

Earlier studies have suggested that a CaSR reduction increases the lymph node metastasis risk in breast cancer. The rs17251221 genotype SNP of CaSR is associated with tumour cell susceptibility and multiple clinicopathological characteristics, including age at diagnosis, tumour size, lymph node metastasis and the oestrogen receptor status of breast cancer tissue^27^. VEGFR3, which is inversely correlated with CaSR, is elevated in endometrial cancer. VEGFR3 may mediate VEGF-C- and VEGF-D-triggered lymphangiogenesis^28^. CaSR activation may suppress lymphangiogenesis in endometrial cancer by restraining VEGFR3 expression, though further evidence is needed.

This study is the first to explore the role of CaSR in endometrial cancer, and further investigations are warranted to clarify the precise mechanism of its inhibitory effect. Our data confirm that the apoptosis that is induced by increased [Ca2+]i is proportional to (but does not completely rely on) CaSR expression and function. Thus, it is worth pursuing whether an adjuvant therapeutic approach for endometrial cancer can be developed based on the regulation of calcium metabolism or by specifically targeting CaSR. Moreover, how the CaSR exerts its physiological and pathological functions in normal uterine and cancerous endometrial tissues remains undetermined.

Our study is also the first to prove that CaSR is expressed in endometrial cancer where it induces apoptosis. Additionally, activated CaSR facilitates E-cadherin/β-catenin complex formation at the cell membranes of endometrial cancer cells to potentially inhibit tumour invasion. These results identify CaSR as a tumour suppressor that may serve as a biological marker for the malignant progression of endometrial cancer. The precise mechanism and therapeutic potential of CaSR in endometrial cancer remains unexplored.

## Materials and Methods

### Cell culture and tissue specimens

The Ishikawa endometrial cancer cell line was obtained from the American Type Culture Collection (ATCC). Cells were cultured in Dulbecco’s modified Eagle’s medium (DMEM, HyClone, USA) supplemented with 10% foetal bovine serum (GIBCO, Invitrogen, USA), 100 U/ml penicillin, 100 μg/ml streptomycin and 1.8 mM Ca2+. Cells were maintained at 37 °C in a 5% CO2 humidified incubator.

Tissue specimens were obtained from patients who had been diagnosed with endometrial cancer (n = 50) and from normal endometria (n = 10) as previously described^29^. All patients had undergone surgical resections or biopsies at Wuhan Union Hospital between 2010 and 2013, and the diagnoses were confirmed by a gynaecologic pathologist. This study was approved by the Ethics Committee of Tongji Medical College, Huazhong University of Science and Technology (IORG no: IORG0003571) after receiving informed consent from the patients. All methods were performed in accordance with the relevant guidelines and regulations.

### Transfections

Effective short-hairpin RNA plasmids (HSH020899-LVRU6GP) that targeted human CaSR (gene ID: 846, GenBank accession number: NM 000388.2) and plasmids that contained the human CaSR cDNA (EX-T8430-Lv105-5) were obtained from GeneCopoenia (Guangzhou, China). They were used to package the lentivirus-delivered CaSR shRNA, control shRNA, and the CaSR overexpression (CaSR OV) or control vectors with the lenti-Pac™ HIV Expression Packaging System (GeneCopoenia HPK-LvTR-20). Ishikawa cells were transiently transfected with the lentivirus per the manufacturer’s instructions as follows: the cells were seeded into a 6-cm culture dish with fresh medium containing the lentivirus (100 μl, 1 × 10^8^ TU/mL) and Polybrene (6 µg/ml) for 8 h until the cell density reached 60–70%. The medium was replaced with fresh medium containing 5% foetal bovine serum for 72 h.

### [Ca2+]i measurement

The transfected Ishikawa cells were seeded onto glass coverslips before the [Ca2+]i measurement. The cells were incubated with 5 μM Fura-2 (Invitrogen-Molecular Probes, F14185) for 30 min at 37 °C in the dark, washed three times and subjected to de-esterification with 4-(2-hydroxyethyl)-1-piperazineethanesulfonic acid-buffered saline (pH 7.40; 5 mM HEPES; 135 mM NaCl; 5.0 mM KCl; 0.07 mM CaCl2; 1.2 mM MgSO4; 10 mM d-glucose) for 30 min. The cells on the coverslips were fixed in a tailor-made groove, which connected them to a sealed perfusion system, and perfused with HBS supplemented with 0.07 mM extracellular Ca2+([Ca2+]o). After 5 min 2.0 mM [Ca2+]o were administered at room temperature. Fura-2, the excitable probe for Ca was excited under an inverted fluorescence microscope (Olympus IX-70). Changes in fluorescence were examined using an Olympus objective lens (Olympus UApo/340 40 × /0.90) and a CCD camera (Qimaging FAST1394, Surrey, BC). The Invivo3 software (Media Cybernetics, Rockville, MD) and Image-pro Analyzer 6.2 (Media Cybernetics) were used to capture and analyse the image, respectively.

### Western blotting

The ProteoExtract® Transmembrane Protein Extraction Kit (TM-PEK) (Novagen, prod#71772-3) was used to extract the β-catenin membrane proteins, and the NE-PER Nuclear and Cytoplasmic Extraction Reagents (Thermo Scientific™, prod#78835) were used to extract the β-catenin nuclear and cytoplasmic proteins, respectively. For the E-cadherin, CASR and VEGFR3 proteins, Ishikawa cells were lysed in 500 μl of a protease inhibitor-containing Pierce™ IP Lysis Buffer (prod#87788). The all above cell lysate was pre-cleared with Pierce™ Protein G Plus Agarose (prod#22851) for 1 h at 4 °C, and the supernatant was collected into a new tube for the protein concentration measurement after centrifugation at 14,000 g for 15 min at 4 °C.The 40 μg/hole protein samples were separated by SDS-PAGE (10%) and incubated with an anti-β-catenin polyclonal antibody (Abcam: ab32572, 1:2000), an anti-E-cadherin antibody (Abcam: ab40772, 1:2000), CASR(6D4) (Santa Cruz Biotechnology: #sc-47741,1:1000) antibody or anti-VEGFR3(Santa Cruz Biotechnology: #sc-20734, 1:1000). The protein quantity was measured using ECL after incubations with horseradish peroxidase (HRP)-labelled species-specific secondary antibodies (1:8000, Affbiotech).

### Co-immunoprecipitation

The total protein lysate (1 mg/500 μl) was incubated with 5 μg mouse monoclonal anti-E-Cadherin antibody on a rotating shaker at 4 °C overnight and mixed with 50 μl of fresh Protein G Plus Agarose for 4 h. The pellet was washed 5 times and resuspended in 50 μl 2 × SDS loading buffer for SDS-PAGE using the anti-β-catenin polyclonal antibody and the anti-E-cadherin antibody. The subsequent Western blotting steps are described above.

### RNA extraction and real-time PCR

RT-PCR assays were conducted to examine the post-transfection mRNA amplification levels of CaSR, E-cadherin and β-catenin in Ishikawa cells. Total RNA was extracted with Trizol agents and reverse transcription-PCR (TAKARA) was performed using 1 μg/μl total RNA as the template. Real-time PCR was conducted in a 20 μl reaction volume that contained 1.6 μl cDNA template, 0.8 μl 5′primer, 0.8 μl 3′primer, and 10 μl 2 × premix ex Taq. The primer sequences were as follows: human CaSR (F: GCTGTTTATCTCCTCTATG; R: GGGCTCTTT CCTATTCAT), human E-cadherin (F: GAGCACCTTCCATGACAGACCC, R: GAGAAGGCATTGACATACAC), β-catenin (F: TGTATGGGTAGGGTAAATCAGTAAG, R: CTCTTGA AGACGTATCACAGC), human β-actin (F: GCTGTCACCTTCACCGTT, R: CTCAT CTGGCCT CGCTGT). The 2−ΔΔCT method was used to calculate mRNA amplification.

### Immunofluorescence

Transfected Ishikawa cells were fixed with a 4% paraformaldehyde solution for 15 min on coverslips, permeabilized in 0.3% TritonX-100 and blocked with 5% bovine serum albumin for 30 min. The cells were incubated with anti-E-cadherin (Abcam: ab40772,1:50) and anti-β-catenin (Abcam: ab32572,1:50) antibody overnight at 4 °C, which were subsequently specifically bound with an anti-mouse IgG-FITC-conjugated secondary antibody (BioVision; 1:1000) and an anti-rabbit IgG-Cy3-conjugated secondary antibody (Jackson; 1:1000) for 30 min at room temperature, respectively. Images were captured with an inverted confocal fluorescence microscope (Olympus IX71) after nuclear staining with 4’,6-diamidino -2-phenylindole.

### Cell viability and apoptosis assays

The CCK8 kit (Sigma) was used to measure the effect of CaSR expression on cell viability. One thousand five hundred transfected Ishikawa cells were cultured in 96-well plates for 24 h and synchronized with FBS-free media for 24 h. Optical densities were measured with a spectrophotometer at 450 nm after 2-h incubations with 10 μl CCK8 solution per well. Cell apoptosis was assessed using a cell death detection ELISA kit (Roche, Palo Alto, CA), which measured the cytoplasmic histone-associated DNA fragmentation that occurred with apoptosis.

### Transwell cell invasion assay

Invasion assays were performed to assess the effect of CaSR expression on the invasiveness of endometrial cancer cell. The transfected Ishikawa cells (1 × 10^5^ cells per well) were seeded into upper chambers that were pre-coated with the Matrigel basement membrane matrix (BD Biosciences, Bedford, MA, USA). The cells were starved in a serum-free medium for 12 h before seeding. Six hundred microliters of 10% foetal bovine serum-containing medium were added into each lower chamber, and cells were maintained at 37 °C in a 5% CO2 humidified incubator for 48 h. After the non-invasive cells were removed, the invasive cells were stained with 0.5% crystal violet and counted in five random fields per slide under a microscope.

### Immunohistochemistry

Normal and cancerous endometrial tissue samples were used to measure CaSR, E-cadherin, β-catenin and VEGFR3 expression. The four-micrometre-thick tissue sections were deparaffinized and rehydrated on slides, and the endogenous peroxidase gradually quenched. Antigen retrieval was performed with sodium citrate buffer (0.01 M, pH 6.0) in a microwave for 15 min. Nonspecific staining was blocked with 5% bovine serum albumin for 30 min. The slides were incubated with the anti-CaSR (Santa Cruz Biotechnology: #sc-47741,1:150), anti-E-cadherin (Abcam: ab40772,1:200), anti- β-catenin (Abcam: ab32572,1:100) and anti-VEGFR3 (1:100; Santa Cruz Biotechnology, #sc-20734) antibodies overnight at 4 °C, followed by anti-rabbit and anti-mouse IgG-Horseradish peroxidase-conjugated secondary antibodies (1:1000) for 30 min at room temperature; visualization was performed using the 3-3′ diaminobenzidine tetrachloride (DAB) substrate. The slides were rinsed, counterstained with haematoxylin, dehydrated in alcohol and hyalinized with xylene. The staining intensity was independently evaluated by two pathologists. The scoring categories (none, 0; weak, 1; moderate, 2; and strong, 3) were multiplied by the percentage of positive tumour cells (<10%, 1; 10–49%, 2; >50%, 3) to calculate the final score. A histological score ≤1 point was defined as negative, and ≥2 points was considered as positive.

### Statistical analysis

The statistical analysis was performed using the SPSS version 17.0 statistical software (Chicago, IL, USA). Values are presented as the mean ± SD. The Student’s t-test and one-way analysis of variance were used for two- and multi-group comparisons, respectively. The software performed multiple comparisons of means using the Least Significant Difference and Student-Newman-Keuls tests, and One-samples Kolmogorov-smirnow test was used to test the normal distribution. P-values that fell below 0.05 (two-tailed) were considered significant.

## Electronic supplementary material

Supplementary Information
