# Supplementary material for: The suppressive role of calcium sensing receptor in endometrial cancer
Source: Sci Rep. 2018 Jan 18;8:1076. doi: 10.1038/s41598-018-19286-1 (PMC5773571; doi:10.1038/s41598-018-19286-1)
Supplement: Supplementary file 1 — Supplementary Information [file 41598_2018_19286_MOESM1_ESM.pdf]

Supplementary information

Title: The suppressive role of calcium sensing receptor in endometrial cancer

Xiaoyan Xin1¶, Xianqin Zeng2¶, Dilu Feng1, Teng Hua1, Shuangge Liu1, Shuqi Chi1, Qinghua Hu2*, Hongbo Wang1*

1. Department of Gynecology and Obstetrics, Union Hospital, Tongji Medical College, Huazhong University of Science and Technology, No.1277, Jiefang Avenue, Jiangan District, Wuhan PR China, 430022.

2. Department of Pathophysiology, School of Basic Medicine, Tongji Medical College, Huazhong University of Science and Technology, No.13, Hangkong Road, Qiaokou District, Wuhan PR China, 430030.

¶ ¶: Xianqin Zeng and Xiaoyan Xin contributed equally to this work.

***Corresponding author:**

Professor Hongbo Wang, Department of Gynecology and Obstetrics, Union Hospital, Tongji Medical College, Huazhong University of Science and Technology, No.1277, Jiefang Avenue, Jiangan District, Wuhan PR China. Tel: +86-02785726301; Fax: +86-02785726301; E-mail: hb_wang1969@sina.com.

Professor Qinghua Hu, Department of Pathophysiology, School of Basic Medicine, Tongji Medical College, Huazhong University of Science and Technology, No.13, Hangkong Road, Qiaokou District, Wuhan PR China, 430030. E-mail: qinghuaa@mails.tjmu.edu.cn.

Supplemental Table S1

Table 1 Clinicopathological characteristics and CaSR expression in patients with endometrial cancer

Parameters	CaSR expression (n=50)	CaSR expression average score	*p* value
Age			
<50	14	7.857	0.1813
≥50	36	7.672	
FIGO stage			
I/II	39	8.056	0.0774
III/IV	11	6.545	
Histologic grade			
G1	26	8.508	**0.0272**
G2/G3	24	6.875	
Myometrial invasion			
<1/2 of myometrium	34	8.212	**0.0482**
≥1/2 of myometrium	16	6.688	
LN metastasis			
Absent	44	8.073	**0.0077**
Present	6	5.167	

FIGO International Federation of Gynecology and Obstetrics

Supplemental Figure S1

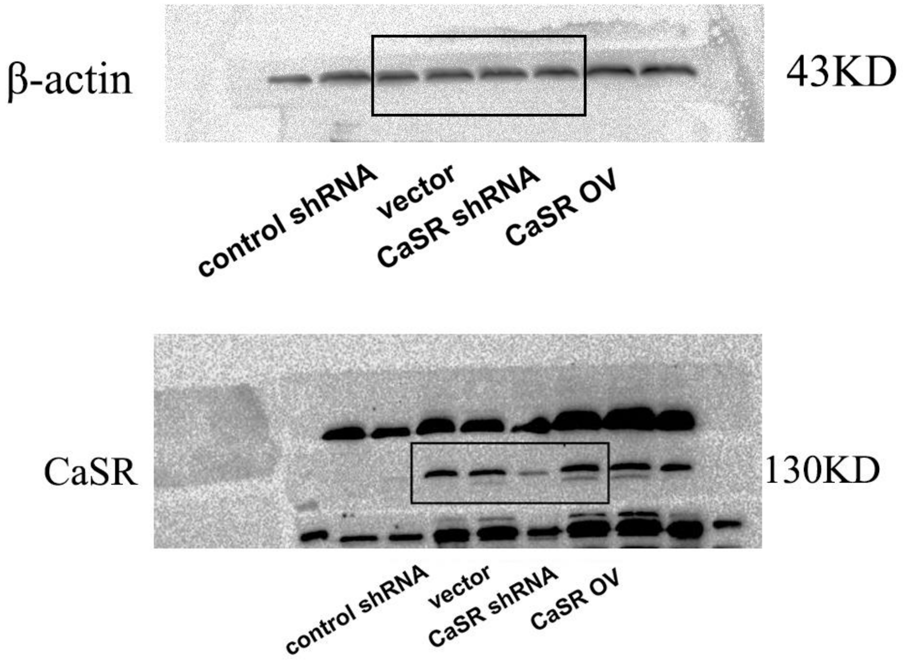

Supplementary Figure S1. Full length blots of Figure 1A. black dotted lines show the cropping locations.

Supplemental Figure S2

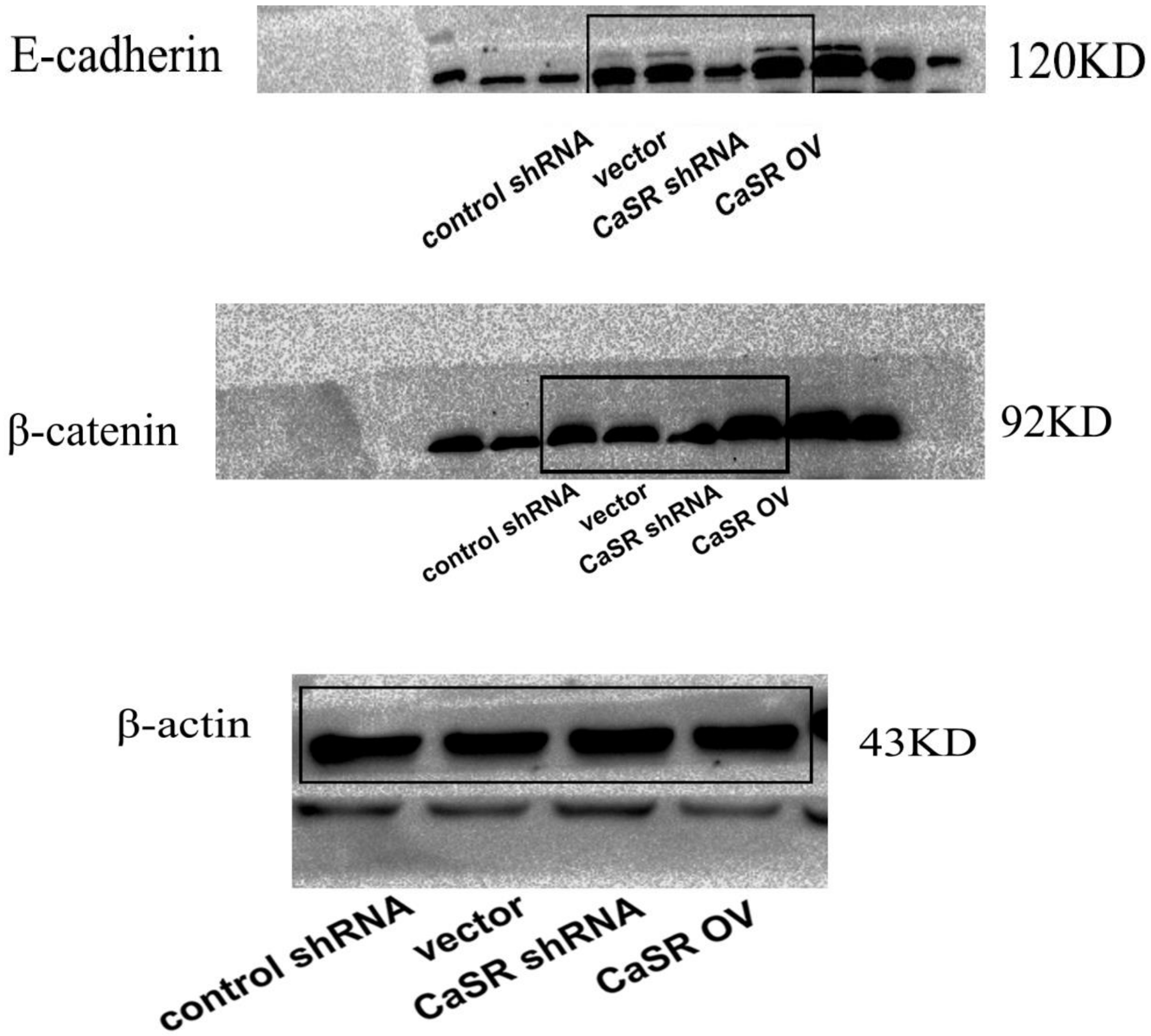

Supplementary Figure S2. Full length blots of Figure 3C. black dotted lines show the cropping locations.

Supplemental Figure S3

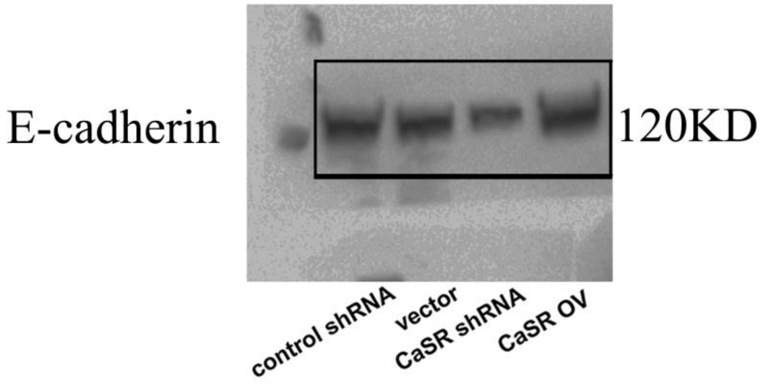

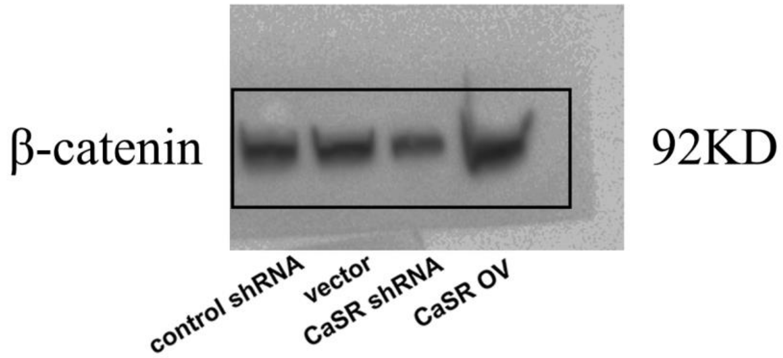

Supplementary Figure S3. Full length blots of Figure 3F. black dotted lines show the cropping locations.

Supplemental Figure S4

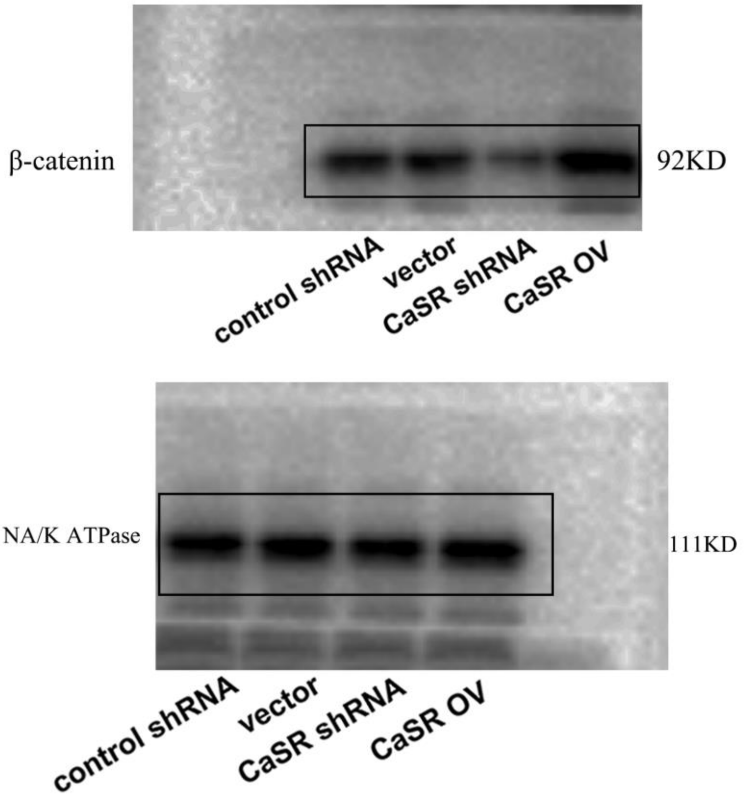

Supplementary Figure S4. Full length blots of Figure 4F. black dotted lines show the cropping locations.

Supplemental Figure S5

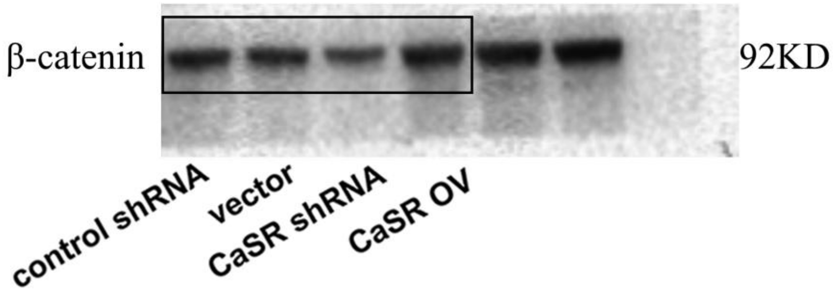

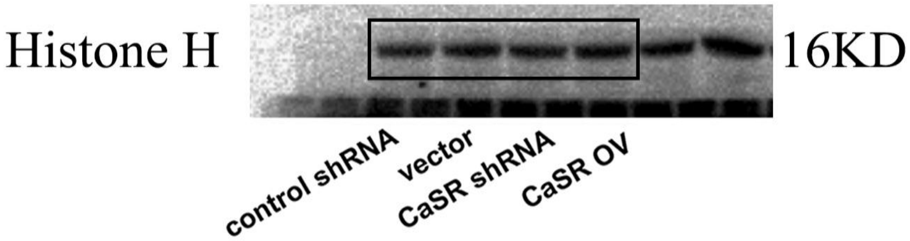

Supplementary Figure S5. Full length blots of Figure 4G. black dotted lines show the cropping locations.

Supplemental Figure S6

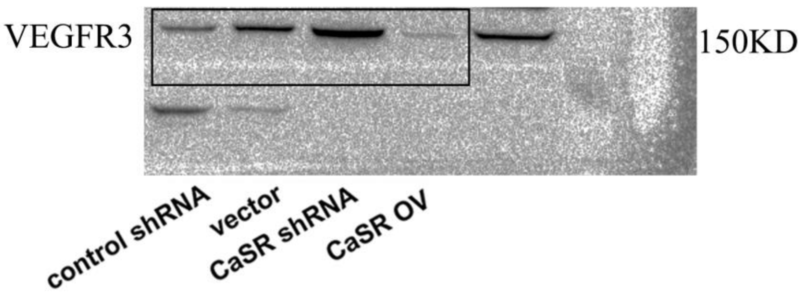

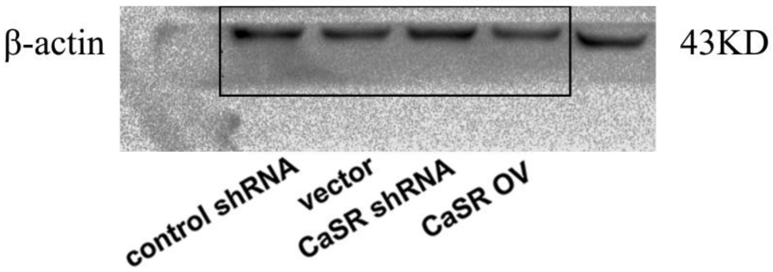

Supplementary Figure S6. Full length blots of Figure 6A. black dotted lines show the cropping locations.
